# Study of the competition between *Colletotrichum godetiae* and *C. nymphaeae*, two pathogenic species in olive

**DOI:** 10.1038/s41598-023-32585-6

**Published:** 2023-04-01

**Authors:** M. Teresa Garcia-Lopez, M. Socorro Serrano, Boris X. Camiletti, Ana Gordon, Cristina Estudillo, Antonio Trapero, Concepcion M. Diez, Juan Moral

**Affiliations:** 1 Department of Agronomy, Maria de Maeztu Excellence Unit, University of Cordoba, Edif. C4, Campus de Rabanales, 14071 Cordoba, Spain; 2 Department of Plant Pathology, University of California-Davis, Kearney Agricultural Research and Extension Center, Parlier, CA 93648 USA

**Keywords:** Fungi, Fungal biology

## Abstract

Olive anthracnose, a critical olive fruit disease that adversely impacts oil quality, is caused by *Colletotrichum* species. A dominant *Colletotrichum* species and several secondary species have been identified in each olive-growing region. This study surveys the interspecific competition between *C. godetiae*, dominant in Spain, and *C. nymphaeae*, prevalent in Portugal, to shed light on the cause of this disparity. When Petri-dishes of Potato Dextrose Agar (PDA) and diluted PDA were co-inoculated with spore mixes produced by both species, *C. godetiae* displaced *C. nymphaeae,* even if the percentage of spores in the initial spore mix inoculation was just 5 and 95%, respectively. The *C. godetiae* and *C. nymphaeae* species showed similar fruit virulence in separate inoculations in both cultivars, the Portuguese cv. Galega Vulgar and the Spanish cv. Hojiblanca, and no cultivar specialization was observed. However, when olive fruits were co-inoculated, the *C. godetiae* species showed a higher competitive ability and partially displaced the *C. nymphaeae* species. Furthermore, both *Colletotrichum* species showed a similar leaf survival rate. Lastly, *C. godetiae* was more resistant to metallic copper than *C. nymphaeae.* The work developed here allows a deeper understanding of the competition between *C. godetiae* and *C. nymphaeae*, which could lead to developing strategies for more efficient disease risk assessment.

## Introduction

The olive tree is the most widely grown woody crop, covering 12 million hectares worldwide, primarily in Mediterranean regions^1^. European olive production accounts for 65% of the world market, with Spain being the primary producer, followed by Italy, Greece, and Portugal^2^. As a result, the olive industry exerts an enormous social and economic impact on Mediterranean countries.

The olive tree can be affected by several diseases and pests. The most important fruit disease of the olive tree is anthracnose, caused by *Colletotrichum* genus fungal species, which share importance with damages caused by the olive fly pest (*Bactrocera oleae* Rossi) since both can lead to significant yield losses^3^ and, also, impact oil quality^4–6^. In Spain, an average of 2.6% of the crop losses country-wide are attributed to anthracnose^4^.

Olive anthracnose presents two differentiated syndromes (i.e., a combination of symptoms or symptoms and pathogen signs): fruit rot and branch dieback. Affected fruits show necrotic lesions at an early ripening stage, which progress until total drupe rot^4,5^. On affected fruits, the fungus develops asexual fruiting bodies (acervuli) that produce a mass of conidia in a pinkish-orange gelatinous matrix^6,7^. As humidity drops and temperature increases, the affected olive fruits mummify^5,7^. The *Colletotrichum* species then colonize the peduncle of affected fruits, which fall to the ground. However, some mummified fruits may remain in the tree canopy until the next fall-winter^7^. In olives affected by the second syndrome, apical leaves show necrotic margins, which may progress until their complete drying, causing shoot and main branch dieback^4,7,8^. This second syndrome has been associated with pathogen toxins produced in affected fruits and mobilized to the shoots^7,9^. Since acervuli development on infected leaves is uncommon, olive canopy mummified fruits are the primary inoculum source^3,5,6^. However, some authors suggest that infected leaves are also an inoculum reservoir^10^. Because *Colletotrichum* species are specialized in infecting olive fruits, species that could infect and remain on the leaves would have an essential competitive advantage^10^.

Anthracnose is widespread and can be found in most olive-growing regions, including non-Mediterranean areas. Eighteen *Colletotrichum* species have been identified as olive anthracnose causal agents around the world^3–5^. These species are clustered into three complexes: *C. acutatum* J.H. Simmonds, *C. gloeosporioides* (Penz.) Penz. & Sacc., and *C. boninense* Moriwaki, Toy. Sato & Tsukib^4,11^. Notably, a dominant *Colletotrichum* species is usually described in each region, while other species of this genus are secondary or rare^6,10,11^. Similarly, every traditional olive-growing country has its own national cultivars, which can be classified according to their importance and dissemination from major cultivars (prevailing in more olive districts) to local cultivars (single olives in a specific district)^12^. Thus, *Colletrotrichum* species and traditional olive cultivars combine in pairs characteristic of the different endemic regions. For example, 'Galega Vulgar' is a representative cultivar in Portugal, being susceptible to the fungus^4,15^. In Portugal, the anthracnose epidemic outbreaks are caused chiefly by the *C. nymphaeae* species^4,11,14^. On the same peninsula, in the anthracnose endemic areas of the Andalusia region (in southern Spain), the highly susceptible 'Hojiblanca', 'Lechin de Sevilla', and 'Picudo' are major cultivars^3^. In Andalusia, *C. godetiae* is the dominant species in olive orchards, just as in Greece, Montenegro, and Italy^5,11,14,25^.

Several authors have described the coexistence between an abundant *Colletotrichum* species accompanied by several secondary species of this genus in olive orchards around the world^11,15,16^. However, the potential mechanisms determining why certain *Colletotrichum* species are dominant over others remain unknown. This unbalanced presence of the *Colletotrichum* species might also be attributed to (1) differences in the capacity of species to produce inoculum in olive leaves, (2) adaptation to the regional climate, (3) a pathogenic specialization in local dominant cultivars, (4) the ability to infect other hosts (e.g., weeds), or (5) a priority effect, i.e., the order of species arrival in the olive orchard can influence the species pattern^10,13,14^. Also, the selection pressure exerted by the recurrent application of copper-based fungicides (the main fungicides used in olive orchards) could also drive the differences among *Colletotrichum* species based on their resistance^17^.

This study seeks to shed light on the unbalanced distribution of *Colletotrichum* species affecting olive trees, focusing on understanding the interspecific competition between *C. godetiae* and *C. nymphaeae* in (1) dual-cultured media of different richness, (2) fruits and leaves of the Portuguese cv. Galega Vulgar and the Spanish cv. Hojiblanca, and (3) olive plants under control conditions.

Through this study, we could elucidate aspects such as the species of the pathogen's cultivar or tissue specialization. Consequently, information extracted from our study provides insights into the *Colletotrichum* spp. population dynamic in olive groves as well as knowledge to improve olive anthracnose disease risk assessment.

## Methods

### Fungal isolates

**Figure 1:**
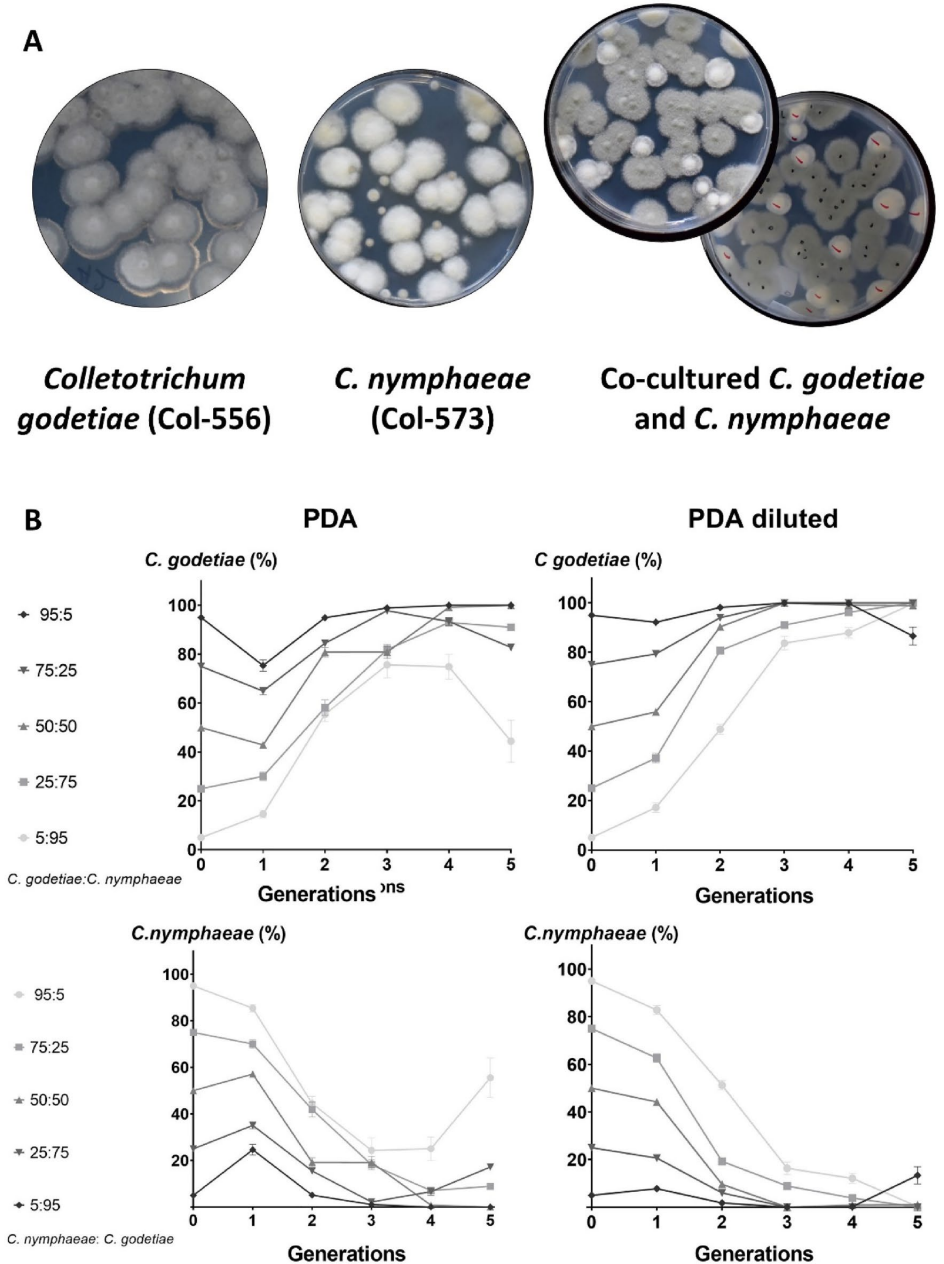
(**A**) Dark grey colonies of *Colletotrichum godetiae* (Col-556) white cottony colonies of *C. nymphaeae* (Col-573) and co-cultured *C. godetiae* (Col-556) vs *C. nymphaeae* (Col-573) on Potato Dextrose Agar (PDA) medium; (**B**) the relative percentage of colonies (Mean and Standard Error values by grouping the three repetitions conducted) of *C. godetiae* and *C. nymphaeae* grown on PDA and diluted PDA co-inoculated with five different initial ratios of conidial mixtures. The treatments correspond to different spore ratio of both *Colletotrichum* species at the beginning of the experiment (generation 1); 95:5; 75:25; 50:50; 25;75; and 5:95 ratios (%) of spores of *C. godetiae*: *C. nymphaeae*.

Different *C. godetiae* and *C. nymphaeae* strains stored at the mycological collection of the Agronomy Department of the University of Cordoba (Spain) were used during various experiments. Two *C. nymphaeae* strains, Col-573 and Col-580, were obtained from affected olive groves in Capela and Montesadinha (Portugal). *C. godetiae* strains Col-556 and Col-703, were obtained from olives that were severely affected by Anthracnose in Beja (Portugal) and Huelva (Spain), respectively. Strains were identified by their internal transcribed spacer 5.8S and β-tubulin regions^11^. Since both *Colletotrichum* species show distinctive morphological characteristics on Potato Dextrose Agar (PDA; 25% potato lixiviated, 2% dextrose, 2% agar, and pH 4.5^18^), these were quickly identified and quantified in dual-culture assays. Overall, *C. godetiae* colonies were dark-grey, while *C. nymphaeae* colonies were white with cottony mycelium in PDA (Fig. 1A). Our assays assume that the interaction between both *Colletotrichum* species involves an interspecific (distinct species) competition for the same resource (media, fruits, or leaves)^19^.

### Conidial suspension preparation

For inoculum production, *Colletotrichum* strains were grown on PDA at 20 °C in darkness for 7 days. Conidial suspensions were individually prepared by scraping pathogen colonies from the medium surface using a scalpel and were transferred to two 250-ml Erlenmeyer flasks containing 100 ml of sterile distilled water. Both resulting suspensions were filtered through a double-layered sterile cheesecloth to remove hyphal fragments. Conidial suspensions were adjusted to a final density of 3 × 10^5^ conidia/ml using a Neubauer chamber.

### Competition on media: effects of culture media and temperature

Dual-culture assays were performed to determine the influence of culture media and temperature on the competitive displacement of one species by another^23^. Three different conidial suspensions (5 × 10^2^ conidia/ml) were prepared: *C. godetiae*, *C. nymphaeae*, and a mixture of both species at the same ratio (50:50). The three types of spore suspension were separately cultured by aseptically spreading 100 µl per 90-mm Petri dishes (50 conidia per dish), with 20 ml of PDA or diluted PDA (4% potato lixiviated, 0.75% dextrose, 2% agar, and pH 4.5). Diluted PDA was included as a medium with a carbon concentration below PDA^18^. Petri dishes were incubated at 15, 20, and 25 °C in darkness. Five replicated dishes per combination of *Colletotrichum* species-conidial suspension-culture medium-temperature were used in a fully randomized design. After a 4-day-growing period, cultures were examined, and the number of colonies of each species per plate was recorded. Then, the percentage of each species relative to the total number of colonies per plate was calculated.

### Competition on media: effect of the initial inoculum

By mixing conidial suspensions of both Col-556 and Col-573 strains, previously prepared as described above, suspensions were prepared to contain percentages of conidia at the following ratios: 100:0, 95:5, 75:25, 50:50, 25:75, 5:95, and 0:100 of *C. godetiae*:*C. nymphaeae*. Mixed-conidial suspensions were adjusted to a final density of 5 × 10^2^ conidia.ml.

Competition assays between the *Colletotrichum* species were conducted in PDA and diluted PDA. Five replicated Petri dishes were used for each conidial suspension and culture medium combination. All Petri dishes were aseptically spread with 100 µl of a conidial suspension and incubated in darkness at 20 °C for 5 days. After incubation, colonies of *C. godetiae* (Col-556 strain) and *C. nymphaeae* (Col-573 strain) were recorded as previously described, and results were expressed as relative percentages. Petri dishes with the *Colletotrichum* colonies were washed using 1 ml of sterile water to obtain a new conidial inoculum for the following cycle. The resulting suspension was adjusted to 5 × 10^2^ conidia/ml using sterile distilled water and transferred to a new set of five Petri dishes. The experiment was completed after five cycles (i.e., conidial transference to medium—incubation—conidia washing and quantification) and was replicated thrice. An additional replicate was conducted using strains Col-703 and Col-580 (*C. godetiae* and *C. nymphaeae*, respectively) to contrast the strains' behavior.

### Competition on fruits: effect of the olive cultivar and temperature

Olive fruits of the Spanish and Portuguese cultivars Hojiblanca and Galega Vulgar were collected from 10-year-old olives planted at the World Olive Germplasm Bank (WOGB) in Cordoba (Spain). Both cultivars are vulnerable to the pathogen^13^. Fruits were harvested at 81–85 phenological growth stage (BBCH scale)^20^ and stored at 4 °C until use. Ms. Pablo Morello, responsible of the WOGB, gave us permission for the collection of olive material necessary for the present study.

Fruits were first washed using running water, surface disinfested by immersion in a 10% solution of commercial bleach (Cl at 50 g/l) in sterile water for 1 min, rinsed with sterile water, and air-dried in a biological safety cabinet for 1 h. Fruits were then placed in humid chambers consisting of 22 × 16 × 10 cm plastic containers (20 fruits per chamber), with 100% relative humidity^40^.

Olive fruits were inoculated by spraying a conidial suspension (1 × 10^5^ conidia/ml; 0.4 ml per fruit) of *C. godetiae*, *C. nymphaeae,* or a mixture (50:50) of conidia of both species to assess the interspecific competition during fruit infection. Humid chambers with inoculated fruits were incubated at 15, 20, or 25 °C. A fully randomized design was performed using 162 humid chambers (2 cultivars × 3 conidial suspensions × 3 incubation temperatures × 3 repetitions).

Disease severity was evaluated weekly using a 0–5 rating system, where 0 = no visible symptoms, 1 = visible symptoms affecting less than 25% of the fruit surface, 2 = visible symptoms affecting 26–50% of the surface, 3 = visible symptoms affecting 51–75% of the surface, 4 = visible symptoms affecting 76–99% of the surface, and 5 = completely rotted fruit (100%), showing the typical pinkish-orange gelatinous pustules. For each replication, severity values (0–5) were used to calculate the disease severity index (DSI) using the following formula: DSI = [(Σni × i)/N] × 100, where *i* means severity (0–5), *ni* stands for the number of fruits with a severity *i*, and *N* is the total number of fruits. Data were used to calculate the relative area under the disease progress curve (rAUDPC) through the trapezoidal integration of DSI values over time^21^.

Following 4 weeks of incubation, 8–10 completely rotted olive fruits (severity = 5) from the three replicated humid chambers were grouped and used as an inoculum source. To this end, affected fruits were transferred to 250-ml Erlenmeyer flasks containing 100 ml of sterile distilled water, which were gently shaken manually for 2 min to wash the inoculum. The resulting conidial suspension was filtered as previously described and adjusted to 1 × 10^5^ conidia/ml for new fruit inoculation, mirroring the nature disease cycle. The experiment was ended after three "disease cycles" and conducted twice. In order to quantify the competitive displacement of one species by another, fruits inoculated with the mixture were transferred to 250-ml flasks at the end of the experiment and washed using 100 ml of sterile water. The conidial suspension (5 × 10^2^ conidia/ml) was then spread onto PDA medium, and the number of colonies of each species was recorded as previously described.

### Survival on detached leaves

Three hundred sixty healthy separate leaves of susceptible cultivars Hojiblanca and Galega Vulgar were harvested from adult trees of the WOGB and inoculated to assess the ability of *C. godetiae* and *C. nymphaeae* to infect this tissue. To that end, leaves (180 per cultivar) were surface disinfested in a 10% commercial bleach solution (Cl at 50 g/l) in sterile water for 1 min and placed into six humid chambers (20 leaves per chamber). Leaves were subsequently inoculated spraying a conidial (5 × 10^2^ conidia/ml, 0.4 ml per leaf) suspension of *C. godetiae*: *C. nymphaeae* at a ratio of 50:50 (spores: spores), as described earlier in the case of fruits inoculation. Again, a completely randomized design was used with 18 humid chambers (2 cultivars × 3 conidial suspensions × 3 repetitions) incubated at 20 °C in darkness. Seven days after inoculation, leaves were again surface disinfested to remove the surface conidia that had not established infection in the leaves. Leaves were later incubated for 5 additional days. After incubation, leaves were cut into 0.5-cm^2^ sections (five per leaf), placed onto PDA dishes, and incubated under the same conditions. After 6 days of incubation, colonies of each isolate grown over the pieces of leaf were quantified, and the percentage of pieces colonized with each species was calculated. The experiment was conducted twice.

### Survival on plant leaves

Three-year-old olive plants of cv. Arbequina, a cultivar that is moderately resistant to *Colletotrichum* spp.^13^, were inoculated with conidial suspensions of *C. godetiae*, *C. nymphaeae*, or a mix of both species to assess their ability to survive on leaves throughout the season. Plants were purchased from a commercial nursery and washed using running water. All fruits were manually removed. Conidial suspensions (5 × 10^2^ conidia/ml) of *C. godetiae*: *C. nymphaeae* at ratios of 100:0, 50:50, and 0:100, were prepared as previously detailed. Plants were inoculated by spraying a conidial suspension using 30 ml per plant. Plants sprayed with sterile water were used as non-inoculated control. Inoculated plants were maintained in a dark growing chamber (20 °C, 100% relative humidity) for 24 h to provide conditions conducive to infection. Plants were then transferred to a greenhouse at 25–30 °C, keeping an inter-treatment spacing of 1 m. Olive plants were subject to drip irrigation as required.

Furthermore, all plants were irrigated (10 mm) every month with overhead sprinklers to simulate disease inoculum dispersion under natural conditions. A completely randomized design with 5 plants (replicates) per treatment (conidial suspension) was used, and the experiment was repeated twice. Leaf samples were taken at monthly intervals for six months and consisted of 25 leaves randomly collected from the five plants of each treatment. All samples were surface disinfested as previously discussed, to remove the remaining inoculum. Finally, the number of leaves colonized by each isolate was determined.

### Resistance to copper

The four *Colletotrichum* strains used in our studies were evaluated for their resistance to copper since most olive anthracnose fungicides are copper-based^4,5^. Thus, agar plugs (7-mm diameter) were collected from the edge of actively growing colonies and placed at the center of 9-cm Petri dishes containing PDA medium amended with CuSO_4_ (100 µl/ml) (Copper sulfate; Merk Lab) or control PDA medium (non-amended medium). Five replicates (Petri dishes) per isolate and treatment were prepared. All dishes, including controls, were incubated at 20 °C in darkness. The diameter of each colony was measured when control colonies reached the dish edge, usually 7 days following incubation. The mycelial growth rate (mm per day) and conidia production rate (conidia per cm^2^) were determined per isolate and replication. To this end, conidia were suspended and rinsed with 2 ml of sterile water, and mycelia were scraped off its surface using a sterile blade. The conidial concentration in the resulting suspensions was assessed using a Neubauer chamber. Lastly, the effect of Cu on mycelial growth and conidia production was calculated as a percentage of inhibition concerning the control treatment^17^.

Additionally, Cu absorption of the two *Colletotrichum* species was determined according to Martins et al.^22^ with modifications. Briefly, three 9-mm agar plugs of each *Colletotrichum* isolate were separately placed inside a 100-ml flask containing 50 ml of PDB (Potato Dextrose Broth) amended with CuSO_4_ (100 µl/ml) or control PDB medium. Flasks were incubated in agitation (130 rpm/min) at 23 °C under light/dark conditions for 7 days. The experiment was replicated thrice. After incubation, mycelium was recovered and dried to constant dry weight at 60 °C on a stove. Dried mycelium was subjected to acidic digestion (6N-HCl) for 24 h in order to dissolve the sample completely. Finally, copper quantification was conducted using Mass Spectrometry and Chromatography (Bruker TQ MS).

All the described procedures were conducted following the European and International guidelines and regulations, including those of the University of (Spain), the European Commission, and Food Agriculture Institution (FAO).

### Statistical analysis

The percentage of *Colletotrichum* colonies forming units on PDA or diluted-PDA mono-cultured or dual-cultured at different temperatures was assessed using a General Linear Model considering ANOVA limitations. Subsequently, treatments were compared using a Tukey test adjusted at *P* < 0.05. In the competition assay in media, means and 95% confidence intervals (CI_95_) were calculated, and t values that did not overlap at the CI_95_ were considered significantly (*P* < 0.05) different^23^. In the fruit inoculation assay, rAUDPC data were arcsin-transformed to satisfy normality and homogeneity of variance assumptions. Subsequently, a general ANOVA was applied using the *Colletotrichum* isolate × incubation-temperature × olive-cultivar × experiment-repetition interaction as the error term. Means were compared according to Tukey's test adjusted at *P* < 0.05. A Chi-square was performed to compare the spore production of each *Colletotrichum* species on co-inoculated fruits. Moreover, a Log-linear model was used to review the effect of the cultivar and *Colletotrichum* (or mix) species on total conidial production by the pathogen on inoculated fruits. In experiments performed with detached leaves, data from each cultivar were analyzed using a Chi-square test. The effect of the *Colletotrichum* species on the mycelial and spore inhibition (%) due to Cu was studied using a Wilcoxon Signed Rank Test. This test was also performed to compare conidium and hyphae sizes among *Colletotrichum* species. Data were analyzed using Statistix 10 (version 10; Tallahassee) and SPSS software (version 25.0; SPSS Inc., Chicago).

## Results

### Fungal isolates

As mentioned above, both *Colletotrichum* species were unequivocally identified as growing in culture media (PDA and diluted PDA), which allowed us to conduct the different assays in our study. Overall, conidia of *Colletotrichum* spp*.* were (8.4)12.5–16.1(18.9) × (3)3.9–5.3(7.4) µm for *C. godetiae* (Col-556) and (6.6)8.7–20.4(29.2) × (2.4)2.5–7.1(9.3) µm *C. nymphaeae* (Col-573). Hyphae sizes ranged from (2.1)2.4 to 4.7(6.0) µm for Col-556, and from (1.7)1.9 to 4.2(4.7) µm for Col-573. However, Wilcoxon Rank Sum Test (*P* > 0.05) yielded no significant differences in fungal structures.

### Competition on media: effects of culture media and temperature

Both *Colletotrichum* species developed colonies differentiated on PDA and diluted PDA. According to the Mixed Effects Model, the *Colletotrichum* species (*P* < 0.001) and incubation temperature (*P* = 0.024) exerted a significant effect on the number of fungal colonies on the media. Conversely, the remaining independent variables (culture media and mono- or co-culturing) and their interactions were not significant (*P* > 0.05). Thus, the *C. nymphaeae* formed significantly more colonies than *C. godetiae* on both media (83.5% vs. 62.9%, respectively). Likewise, both fungal species developed more colonies at 20 °C than 15 °C, irrespective of the culture media used. No significant effect (synergism or antagonism, *P* = *0.666*) of the dual-culture of both species was observed (Table 1).

**Table 1:** Percentage of colony-forming units of the species *Colletotrichum godetiae* and *C. nymphaeae* on Potato Dextrose Agar (PDA) or Diluted PDA mono-cultured or dual-cultured and incubated at different temperatures.

	15 °C		20 °C		25 ºC		Average
	Mono-culture^a^	Dual-culture^b^	Mono-culture	Dual-culture	Mono-culture	Dual-culture	
PDA							
*C. godetiae*	65.2	57.6	63.2	56.8	62.8	62.4	61.3B
*C. nymphaeae*	68.0	74.4	100.0	72.8	89.2	81.6	81A
Average	66.3b		73.2ab		74b		71.6
Diluted-PDA							
*C. godetiae*	57.2	56.0	57.2	77.6	59.6	79.2	64.5B
*C. nymphaeae*	83.2	77.6	89.2	98.2	90.4	77.6	86.0A
Average	68.5b		80.5a		76.7ab		75.2

^a^Nine-cm Petri dishes with PDA or diluted-PDA prepared according to Sinclair and Dhingra (1995) were cultured with 50 conidia per dish of *C. godetiae*, *C. nymphaeae*, or a conidial mixture of both species (50:50%)^b^.

### Competition on media: effect of the initial inoculum

Figure 1B represents the percentage of *C. godetiae* and *C. nymphaeae* colonies recovered from dual-cultured PDA and diluted PDA. Considering the global data set, including the three repetitions, overall, the strain Col-556 of *C. godetiae* displaced the strain Col-573 of *C. nymphaeae* in co-inoculated PDA dishes, gradually prevailing over the population regardless of the initial conidial ratio. In PDA dishes, for example, the percentage of *C. godetiae* colonies at the last cycle was over 80% in all the treatments, except for mixture *C. godetiae*: *C. nymphaeae* 05:95, which was 44.43%. Accordingly, a marked prevalence of *C. godetiae* was observed in experiments performed on diluted PDA. In fact, the *C. godetiae* strain was the majority strain on all mixture treatments on completion of the experiment. In the extra replicate that we conducted in diluted PDA using the Col-703 and Col-580 strains of *C. godetiae* and *C. nymphaeae*, respectively, we found the same pattern, with the number of colonies of *C. godetiae* being significantly higher than those of *C. nymphaeae* at the end of the experiment for any combination used (Fig. 1S).

### Competition on fruits: effect of the olive cultivar and temperature.

#### Disease severity

**Figure 2:**
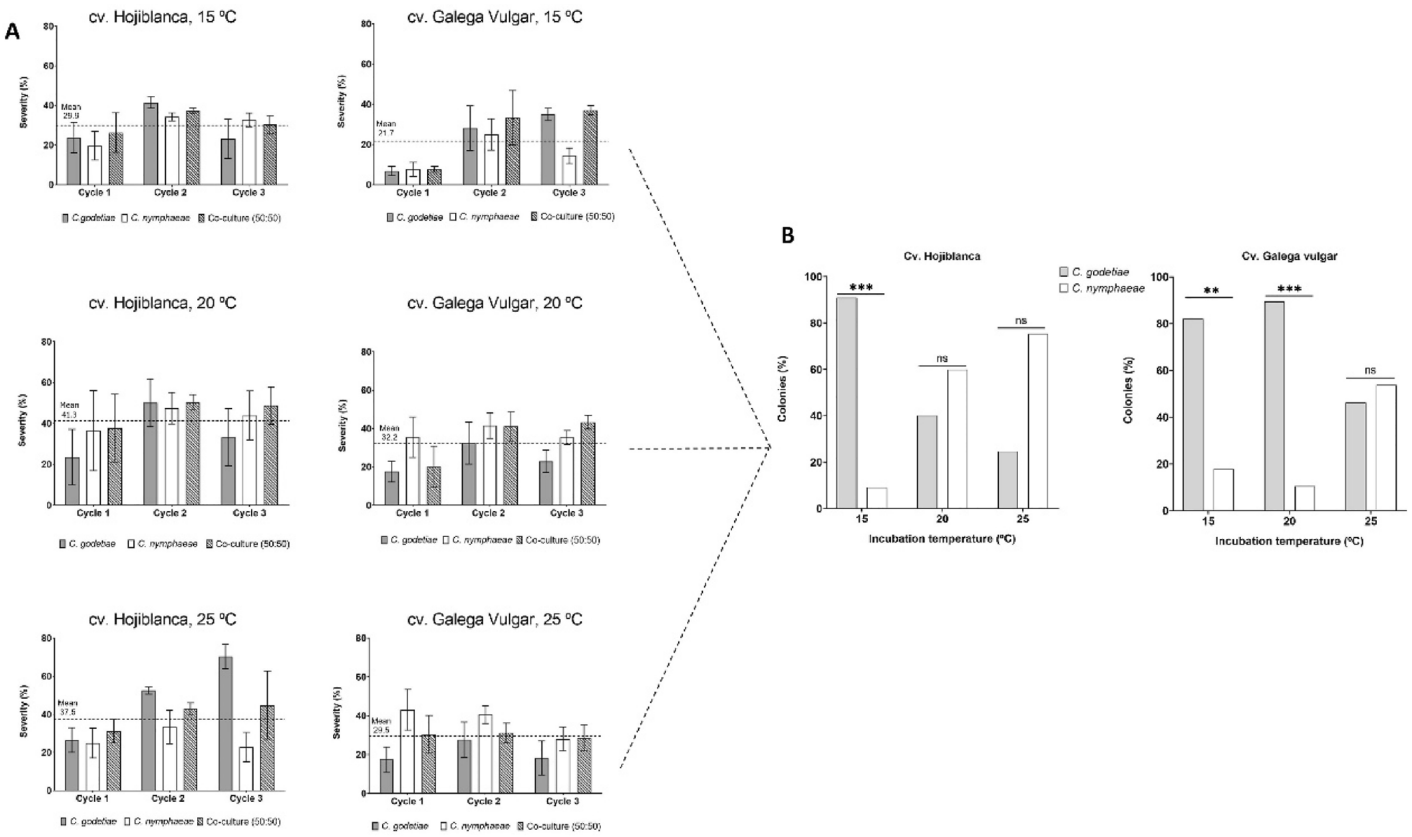
(**A**) Disease severity (Mean and Standard Deviation values by grouping the two repetitions conducted), evaluated as the relative Area Under the Disease Progress Curve (rAUDPC), of olive fruits of cultivars Hojiblanca and Galega Vulgar inoculated with conidial suspensions of *Colletotrichum godetiae*, *C. nymphaeae*, and a mixture of both species (50:50), and incubated at 15, 20 and 25 °C. (**B**) Colonies (%) of *C. godetiae* and *C. nymphaeae* from affected olive fruits of cvs. Hojiblanca and Galega Vulgar co-inoculated with a mix (50:50%) of spores of both species and incubated at 15, 20 and 25 °C. Significant differences between *Colletotrichum* species according to Chi-Square test at *P* < 0.001***,* P* < 0.01**, and *P* < 0.05*.

Disease severity on fruits was separately monitored at every cycle for each combination of cultivar and temperature (Fig. 2A) by grouping the two repetitions conducted since these showed similar patter. Interestingly, the *C. godetiae* and *C. nymphaeae* species showed similar (*P* = 0.501) virulence on the fruits of both olive cultivars. Likewise, our data showed no significant interaction between *Colletotrichum* species and cultivar (*P* = 0.527); i.e., no cultivar pathogenic specialization occured. Furthermore, 'Hojiblanca' fruits were more vulnerable (*P* = 0.032) to the pathogen than 'Galega Vulgar' fruits. Inoculated fruits incubated at 15 °C were less affected (*P* = 0.045) than those set at 25 °C but like those at 20 °C.

#### Quantification of the inoculum produced by each Colletotrichum species in co-inoculated fruits

Our data showed that the species *C. godetiae* displaced its competitor at 15 °C on the inoculated fruits of both cultivars, after three simulated cycles of disease. Likewise, *C. godetiae* was more competitive at 20 °C but only in 'Galega Vulgar' fruits. No significant differences were observed when fruits were incubated at 25 °C (Fig. 2B).

#### Sporulation of C. godetiae and C. nymphaeae in inoculated fruits

When we evaluated the number of spores produced by the affected olive fruits following the Pearson chi-square of the Log-linear model, the total inoculum per fruit was not affected by cultivar *Colletotrichum* species or temperature (Fig. 2S).

### Survival on detached leaves

**Figure 3:**
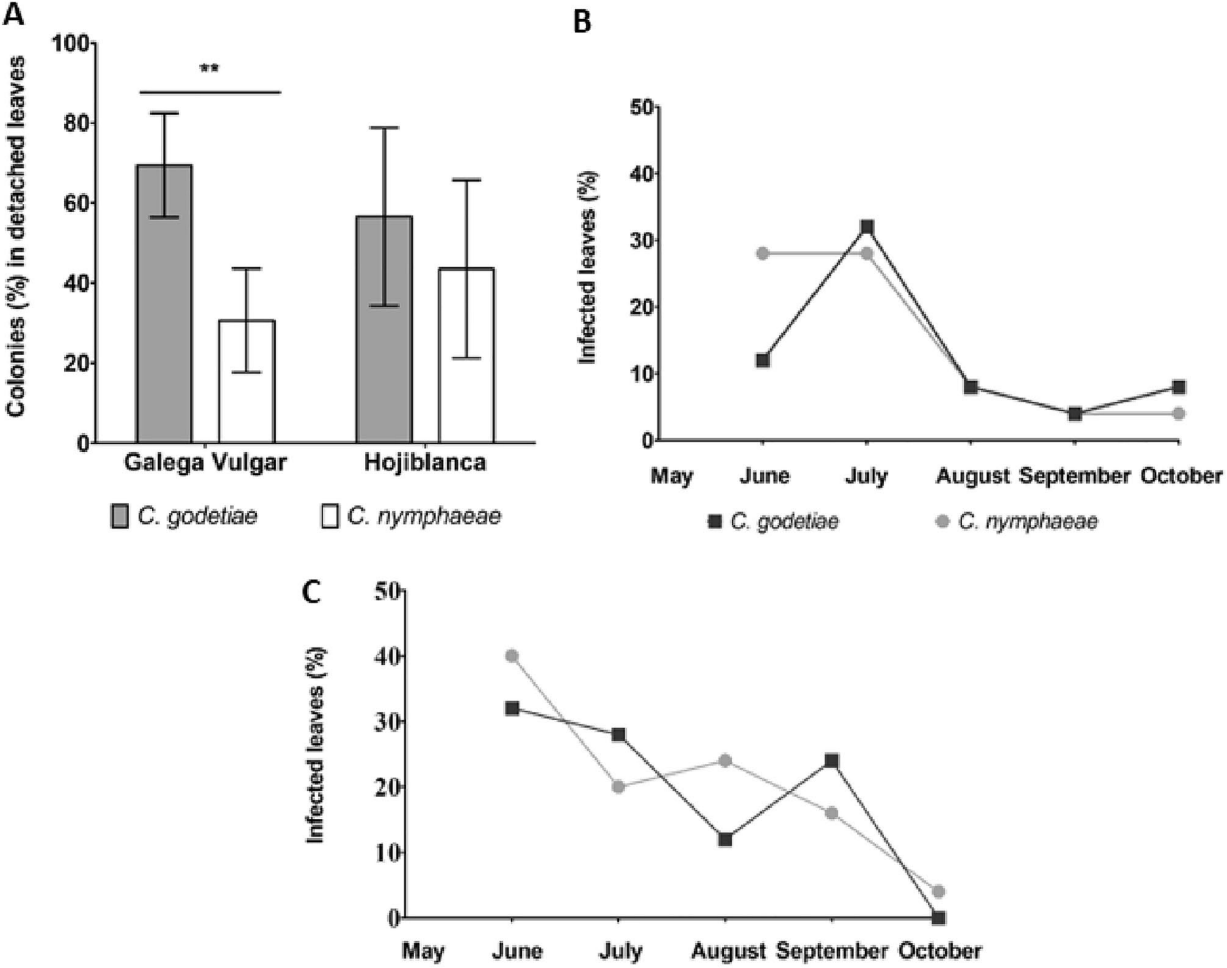
(**A**) Relative percentage of colonies of *Colletotrichum godetiae* and *C. nymphaeae* recovered from detached leaves of cultivars Galega Vulgar and Hojiblanca co-inoculated with both isolates. Percentage of olive leaves cv. Arbequina, which were co-inoculated (**B**) or independently inoculated (**C**) with both *Colletotrichum* species, the pathogens of which were recovered. *Significance between relative percentages according to the Chi-Square test.

When olive leaves cv. Galega Vulgar and Hojiblanca were co-inoculated with both *Colletotrichum* species; *C. godetiae* showed a higher capacity than *C. nymphaeae* to settle in 'Galega Vulgar' leaves (*P* < 0.01), but these differences were not recorded (*P* > 0.05) on the cv. Hojiblanca leaves (Fig. 3A).

### Survival on plant leaves

Overall, *Colletotrichum* strains of both species were recovered from olive leaves of potted cv. Arbequina plants throughout the experiment (Fig. 3B,C). However, a gradual decline in the percentage of leaves colonized by the *Colletotrichum* isolates throughout both experiments occurred, i.e., when plants were separately inoculated with both species or co-inoculated. At the end of both experiments (5 months following inoculation), *Colletotrichum* was isolated from co-inoculated leaves at percentages lower than 10%, but *C. godetiae* species were not isolated from plant leaves that were inoculated only with this species.

### Resistance to copper

*Colletotrichum godetiae* strains were more copper-tolerant (*P* < 0.05) than *C. nymphaeae* strains, according to the percentages of inhibition of mycelial growth (− 0.6 vs. 4.4%) and spore production (− 48.8 vs. 68.8%) when culture media was amended with copper, calculated in relation to an unamended control. In some repetitions (Petri dishes), copper presence induced spore production, mainly on *C. godetiae* strains. Besides, *C. godetiae* resistance to copper was because of its ability to prevent the Cu accumulation in the mycelium (avg. 364 µg Cu/g mycelium lower than *C. nymphaeae*). However, no significant differences (*P* = 0.173) were detected between both species. In any case, colonies of both species growing in Cu-amended media showed significantly higher Cu content than control colonies, reaching concentrations over 100 times denser (Table 2).

**Table 2:** Average values and standard errors of the inhibitions (%) of mycelial growth and spore production; and Cu content (µg/g) in mycelium of both *Colletotrichum godetiae* and *C. nymphaeae*, growing in Potato Dextrose Agar media Cu-amended (100 µl/ml) and unamended (control).

Species	Strains	Inhibition (%)*		Cu concentration (µg/g)	
		Mycelial growth	Spore production	Control	Cu
*C. nymphaeae*	676	3.34 ± 5.1	50.78 ± 17.75	21.8 ± 5.1	2964.7 ± 264.2
	677	5.51 ± 1.4	86.80 ± 4.63	21.9 ± 3.9	2657.7 ± 374.9
*C. godetiae*	715	− 1.21 ± 1.6	− 82.43 ± 82.64	22.8 ± 5.0	2469.0 ± 180.7
	722	− 0.03 ± 1.04	− 15.20 ± 39.88	24.0 ± 5.3	2425.0 ± 156.1

*Negative inhibition (%) values correspond to non-inhibited (i.e., induction) mycelial growth or spore production due to the CuSO_4_ treatment regarding the untreated control.

## Discussion

The olive tree is one of the most widespread woody crops around the world, and demand for olive oil continues to rise^1,12^. Olive anthracnose, caused by several *Colletotrichum* species, is of great concern to olive oil producers because of (1) its negative impact on oil quality^3,5^, (2) its penchant for developing in high plant density, the greatest exponent which is hedge-shaped olive groves, especially conducive to anthracnose development^24^ and (3) the Cu compounds use challenges such as the low sensitivity of the pathogen to copper-based fungicides^17^, and the EU restrictions on the use of plant protection products, to a maximum rate of 4 kg Cu per hectare and year^25^.

The interaction among phytopathogenic fungal species and antagonistic microorganisms (e.g., biological control agents) has triggered the development of biological control in many crop diseases^26^, including the olive anthracnose^27,28^. In contrast, interaction among phytopathogenic agents affecting a given host is much less known^29,30^. In the case of olive anthracnose, the interaction between pathogenic species is of interest for two main reasons: first, because up to 18 *Colletotrichum* species have been associated with the diseases; and second, because in most olive-growing regions, a dominant species coexists with several minority ones ^6,10,11^. For example, *C. godetia*e is the dominant species in Greece, Italy, and Spain, where it coexists with other species, including *C. nymphaeae*, which are much less abundant^11^. The opposite pattern is observed in Portugal, where *C. nymphaeae* is dominant, while *C. godetiae* is only occasionally detected^4,8,11^.

Many ecological reasons might account for the presence of a dominant *Colletotrichum* species in a given olive-growing region, including (1) a high pathogenic specialization on the major cultivar^31^, (2) better adaptability to agroclimatic conditions^24,31^, or (3) a high ability to infect and remain in olive leaves^7,10^. Therefore, assays have been performed in our study to clarify the differential prevalence of *C. godetiae* and *C. nymphaeae* in Spain and Portugal. Fortunately, the morphological features of the colonies in culture media allowed the distinction between both species.

Firstly, competition between *C. godetiae* and *C. nymphaeae* in Petri dishes was stimulated in a dual-cultured assay. Overall, *C. godetiae* displaced its competitor even when a low proportion of spores of this species (5 vs 95%) were initially transferred to two media with different carbon concentrations (PDA and diluted PDA) ^32,33^. Differences in the capacity to assimilate carbon sources among *Colletotrichum* fungal species are well-known and have been used in taxonomic proposals^34^.

Subsequently, we inoculated two major Portuguese and Spanish olive cultivars ('Galega Vulgar' and 'Hojiblanca') with the *C. nymphaeae and C. godetiae* species to explore a potential cultivar specialization (i.e., significant interaction cultivar × specie). However, against expectations, no cultivar specialization was detected, and both species showed similar virulence. In addition, fruit inoculations with both species did not increase or decrease the severity of anthracnose symptoms, i.e., no synergistic or antagonistic effects occurred^35^. Even if it was a co-inoculated fruit, *C. godetiae* competitively displaced *C. nymphaeae*, but it was a partial displacement (not an exclusion) and cultivar and incubation temperature dependent.

Furthermore, inoculated olive fruits underpinned the difference in susceptibility to both *Colletotrichum* species of the cv. Galega Vulgar (susceptible) and cv. Hojiblanca (highly susceptible), previously classified under natural infection conditions ^11^. The absence of *Colletotrichum* species × cultivar interaction is essential for olive-breeding programs since breeding can be directed to a broad range of pathogen species. For example, Talhinhas et al. showed that the Italian cultivar Frantoio is highly resistant to several *Colletotrichum* species^36^. This is even noticeable in new olive genotypes with 'Frantoio' pedigree^37^.

Different *Colletotrichum* species can affect different organs in a single host, as happens with the orange tree, in which *C. acutatum* infects flowers and harvest the syndrome known as post-bloom fruit drop. In turn, *C. gloeosporioides* causes fruit necrosis during ripening and subsequent orange storage thereof^38^. In Spain, our field and experiment data point to a specialization by *C. godetiae* in infecting olive fruit, while leaves play a minimal role as an inoculum source since the pathogen does not develop acervuli in these tissues in the field (acervuli can only be observed in inoculated leaves after a long period of incubation under artificial moisture conditions, *J. Moral personal observation*)^7^. Conversely, infected leaves are regarded as a secondary inoculum source in other countries^10,39^. In any case, the survival capacity of *Colletotrichum* for settling in olive leaves provides a substantial competitive advantage for the pathogen^10^. In our study, the ability of *C. godetiae* species to establish in detached olive leaves was higher than that of *C. nymphaeae* species. Concerning pathogen survival in olive leaves over time, both fungal species achieved steady infections in 30% of leaves, but this rate declined gradually during the season. This points again toward the relatively low importance of infected leaves as an inoculum source for the next season, particularly when compared with the mummified fruit, in which the pathogen produces millions of spores^40^. Remarkably, no *Colletotrichum* acervuli were observed in any inoculated leaves or plants. In any case, no substantial differences between *C. godetiae* and *C. nymphaeae* demonstrate the prevalence of one species over the other.

The competitive ability of a given *Colletotrichum* species is enhanced by its resistance to major fungicides in olive orchards (i.e., copper-based compounds)^3–5^. Here, based on the inhibitions on mycelial growth and spore production due to Cu-amendments, we have demonstrated that *C. godetiae* strains are less affected by this compound than those of *C. nymphaeae*. Unlike other studies, in which resistant fungal strains show a higher ability to store Cu in their mycelia^22^, the *C. godetiae* strains of our study tended to accumulate less Cu than those of *C. nymphaea*. We previously described differences in Cu resistance between both species regarding spore germination in the presence of this compound^17^. While the recurrent use of copper-based fungicides can provide powerful selection pressures, no pesticide applications are available for the olive orchards from which strains were collected. Also, we selected orchards with relatively young plants (6–15 years), excluding *Colletotrichum* strains from ancient orchards where there is an accumulation process of copper^41^. In any case, copper-based fungicides present a multi-site activity; therefore, there is a lower risk of pathogens developing Cu resistances^42^.

In short, we have developed an efficient protocol for simulating interaction cycles between *Colletotrichum* species for competitive studies. Our results showed that *C. godetiae* strains tended to shift (no complete exclusion) those of *C. nymphaeae* on both culture media and olive fruit. Classical coexistence theory argues that species coexistence in a niche cannot occur if species compete for a single limiting resource (reviewed by Mittelbach and McGill^43^) according to the competitive exclusion principle^44^. While the coexistence of both *Colletotrichum* species in each olive orchard has been well-described^10,11,14^, competitive interactions among these species could mainly occur at the individual tree level, not at the grove level since the anthracnose epidemic in a given tree is primarily driven by self-infection process inside of the tree canopy^24^. Also, olive fruits cannot be considered a limiting resource for the pathogen since healthy fruits are abundant, even under the most conducive conditions for the disease^17^. In any case, the coexistence of competitive fungal species is well-known, and it is allowed under different mechanisms despite competition for a single resource^45,46^. In our pathosystems, for example, a priority effect (i.e., original colonizers are challenging to displace) could account for the coexistence of both fungal especies^47,48^. Moreover, other characteristics making a *Colletotrichum* species a better competitor (e.g., the ability to infect weeds) are yet to be studied. Lastly, we do not rule out that the *Colletotrichum* population affecting olives could change because of agronomical and climatic factors, as suggested^15^. Further insights into *Colletotrichum* communities in olive groves could be a powerful tool for identifying population changes and selecting appropriate fungicides.

## Supplementary Information

Supplementary Figure 1.

Supplementary Figure 2.

## Data Availability

The datasets generated during and/or analyzed during the current study are available from the corresponding author upon reasonable request.

## References

[1] IOC. http://www.Internationaloliveoil.Org/. Accessed 5 May 2022.

[2] FAOSTAT. 2018. Crop statistics. FAO, Rome. http://www.fao.org/faostat/en/#data/QC. Accessed 5 May 2022.

[3] Moral J, Xaviér C, Roca LF, Romero J, Moreda W, Trapero A (2014). La Antracnosis del olivo y su efecto en la calidad del aceite, Olive Anthracnose and its effect on oil quality. Grasas y Aceites..

[4] Talhinhas P, Loureiro A, Oliveira H (2018). Olive anthracnose: A yield- and oil quality-degrading disease caused by several species of *Colletotrichum* that differ in virulence, host preference and geographical distribution. Mol. Plant Pathol..

[5] Cacciola SO, Faedda R, Sinatra F, Agosteo GE, Schena L, Frisullo S, di San Lio GM (2012). Olive anthracnose. J. Plant Pathol..

[6] Moreira V, Mondino P, Alaniz S (2021). Olive anthracnose caused by *Colletotrichum* in Uruguay: Field symptoms, species diversity and flowers and fruits pathogenicity. Eur. J. Plant Pathol..

[7] Moral J, De Oliveira R, Trapero A (2009). Elucidation of the disease cycle of olive anthracnose caused by *Colletotrichum acutatum*. Phytopathology.

[8] Moral J, Trapero A (2009). Assessing the susceptibility of olive cultivars to anthracnose caused by *Colletotrichum acutatum*. Plant Dis..

[9] Ballio, A., Bottalico, A., Buonocore, V., Carilli, A., Di Vittorio, V. & Graniti, A. (1969) Mediterranean Phytopathological Union Firenze University Press Production and isolation of aspergillomarasmin B (lycomarasmic acid) from cultures of *Colletotrichum gloeosporioides* Penz. (*Gloeosporium olivarum* Aim.) Vindice Di Vittorio and Antonio Gra. *Firenze Univ. Press Behalf Mediterr. Phytopathol. Union*. **8**, 187–196.

[10] Talhinhas P, Mota-Capitão C, Martins S, Ramos AP, Neves-Martins J, Guerra-Guimarães L, Várzea V, Silva MC, Sreenivasaprasad S, Oliveira H (2011). Epidemiology, histopathology and aetiology of olive anthracnose caused by *Colletotrichum acutatum* and *C. gloeosporioides* in Portugal. Plant Pathol..

[11] Moral J, Agusti-Brisach C, Raya MC, Jurado-Bello J, Lopez-Moral A, Roca LF, Chattaoui M, Rhouma A, Nigro F, Sergeeva F, Trapero A (2021). Diversity of *Colletotrichum* species associated with olive anthracnose worldwide. J. Fungi.

[12] Diez CM, Trujillo I, Martinez-Urdiroz N, Barranco D, Rallo L, Marfil P, Gaut BS (2015). Olive domestication and diversification in the Mediterranean Basin. New Phytol..

[13] Moral J, Xavier CJ, Viruega JR, Roca LF, Caballero J, Trapero A (2017). Variability in susceptibility to anthracnose in the world collection of olive cultivars of Cordoba (Spain). Front. Plant Sci..

[14] Materatski P, Varanda C, Carvalho T, Dias AB, Doroteia Campos M, Rei F, Do Rosário Félix M (2018). Diversity of *Colletotrichum* species associated with olive anthracnose and new perspectives on controlling the disease in Portugal. Agronomy.

[15] Schena L, Abdelfattah A, Mosca S, Li Destri Nicosia MG, Agosteo GE, Cacciola SO (2017). Quantitative detection of *Colletotrichum godetiae* and *C. acutatum sensu stricto* in the phyllosphere and carposphere of olive during four phenological phases. Eur. J. Plant Pathol..

[16] Chattaoui M, Raya MC, Bouri M, Moral J, Perez-Rodriguez M, Trapero A, Msallem M, Rhouma A (2016). Characterization of a *Colletotrichum* population causing anthracnose disease on Olive in northern Tunisia. J. Appl. Microbiol..

[17] Moral J, Agusti-Brisach C, Agalliu G, de Oliveira R, Perez-Rodriguez M, Roca LF, Romero J, Trapero A (2018). Preliminary selection and evaluation of fungicides and natural compounds to control olive anthracnose caused by *Colletotrichum* species. Crop Prot..

[18] Sinclair J.B. & Dhingra, O.D. *Basic Plant Pathology Methods*, Press., C., Ed. (1995).

[19] Begon M, Townsend CR (2020). Ecology: From Individuals to Ecosystems.

[20] Sanz-Cortés F, Martinez-Calvo J, Badenes ML, Bleiholder H, Hack H, Llacer G, Meier U (2012). Phenological growth stages of olive trees (*Olea europaea*). Ann. Appl. Biol..

[21] Moral J, Bouhmidi K, Trapero A (2008). Influence of fruit maturity, cultivar susceptibility, and inoculation method on infection of olive fruit by *Colletotrichum acutatum*. Plant Dis..

[22] Martins F, Soares ME, Oliveira I, Pereira JA, de Lourdes Bastos M, Baptista P (2012). Tolerance and bioaccumulation of copper by the entomopathogen *Beauveria bassiana* (Bals.-Criv.) Vuill. exposed to various copper-based fungicides. Bull Environ. Contam. Toxicol..

[23] Glantz SA (2002). Primer of Biostatistics.

[24] Moral J, Jurado-Bello J, Sánchez MI, Oliveira RD, Trapero A (2012). Effect of temperature, wetness duration, and planting density on olive anthracnose caused by *Colletotrichum* spp. Phytopathology.

[25] Authority European Food Safety (2018). Peer review of the pesticide risk assessment of the active substance copper compounds copper(I), copper(II) variants namely copper hydroxide, copper oxychloride, tribasic copper sulfate, copper(I) oxide. Bordeaux mixture. EFSA J..

[26] Maloy O (1993). Plant Disease Control: Principles and Practice.

[27] Nigro F, Antelmi I, Labarile R, Sion V, Pentimone I (2018). Biological control of olive anthracnose. Acta Horticul..

[28] Preto G, Martins F, Pereira JA, Baptista P (2017). Fungal community in olive fruits of cultivars with different susceptibilities to anthracnose and selection of isolates to be used as biocontrol agents. Biol. Control.

[29] Griffin AS, West SA, Buckling A (2004). Cooperation and competition in pathogenic bacteria. Nature.

[30] Sommerhalder RJ, McDonald BA, Mascher F, Zhan J (2011). Effect of hosts on competition among clones and evidence of differential selection between pathogenic and saprophytic phases in experimental populations of the wheat pathogen *Phaeosphaeria nodorum*. BMC Evol. Biol..

[31] Konno M, Iwamoto S, Seiwa K (2011). Specialization of a fungal pathogen on host tree species in a cross-inoculation experiment. J. Ecol..

[32] Landum MC, Félix M, Alho J, Garcia R, Cabrita MJ, Rei F, Varanda CMR (2016). Antagonistic activity of fungi of *Olea europaea* L. against *Colletotrichum acutatum*. Microbiol. Res..

[33] Nuangmek W, McKenzie EH, Lumyong S (2008). Endophytic fungi from wild banana (*Musa acuminata* Colla) works against anthracnose disease caused by *Colletotrichum musae*. Res. J. Microbiol..

[34] Barnett, H.L. & Hunter, B.B. Illustrated genera of imperfect fungi. in *The American Phytopathological Society*, **200**. Minnesota., Ed. (1998).

[35] Dutt A, Andrivon D, Le May C (2022). Multi-infections, competitive interactions, and pathogen coexistence. Plant Pathol..

[36] Talhinhas P, Gonçalves E, Sreenivasaprasad S, Oliveira H (2015). Virulence diversity of anthracnose pathogens (*Colletotrichum acutatum* and *C. gloeosporioides* species complexes) on eight olive cultivars commonly grown in Portugal. Eur. J. Plant Pathol..

[37] Moral J, Alsalimiya M, Roca LF, Díez CM, Leon L, De la Rosa R, Rallo L, Barranco D, Trapero A (2015). Relative susceptibility of new olive cultivars to *Spilocaea oleagina*, *Colletotrichum acutatum*, and *Pseudocercospora cladosporioides*. Plant Dis..

[38] Zulfiqar M, Brlansky RH, Timmer LW (1996). Infection of flower and vegetative tissues of citrus by *Colletotrichum acutatum* and *C. gloeosporioides*. Mycologia.

[39] Sergeeva V, Spooner-hart R (2008). First report of *Colletotrichum acutatum* and *C. gloeosporioides* causing leaf spots of olives (*Olea europaea*) in Australia. Australas. Plant Dis. Notes.

[40] Moral J, Trapero A (2012). Mummified fruit as a source of inoculum and disease dynamics of olive anthracnose caused by *Colletotrichum* spp. Phytopathology.

[41] Mackie KA, Müller T, Kandeler E (2012). Remediation of copper in vineyards—A mini review. Environ. Poll..

[42] Brent KJ, Hollomon DW (2007). Fungicide Resistance in Crop Pathogens: How Can it be Managed?.

[43] Mittelbach GG, McGill BJ (2019). Community Ecology.

[44] Gause GF (1934). The Struggle for Existence.

[45] Kennedy P (2010). Ectomycorrhizal fungi and interspecific competition: Species interactions, community structure, coexistence mechanisms, and future research directions. New Phytol..

[46] Hiscox J, O’leary J, Boddy L (2018). Fungus wars: Basidiomycete battles in wood decay. Stud. Mycol..

[47] Chesson P (2000). Mechanisms of maintenance of species diversity. Annu Rev. Ecol. Evol. Syst..

[48] Hiscox J, Savoury M, Müller CT, Lindahl BD, Rogers HJ, Boddy L (2015). Priority effects during fungal community establishment in beech wood. ISME J..

