# Supplementary figures and images for: Study of the competition between Colletotrichum godetiae and C. nymphaeae, two pathogenic species in olive
Source: Sci Rep. 2023 Apr 1;13:5344. doi: 10.1038/s41598-023-32585-6 (PMC10067957; doi:10.1038/s41598-023-32585-6)

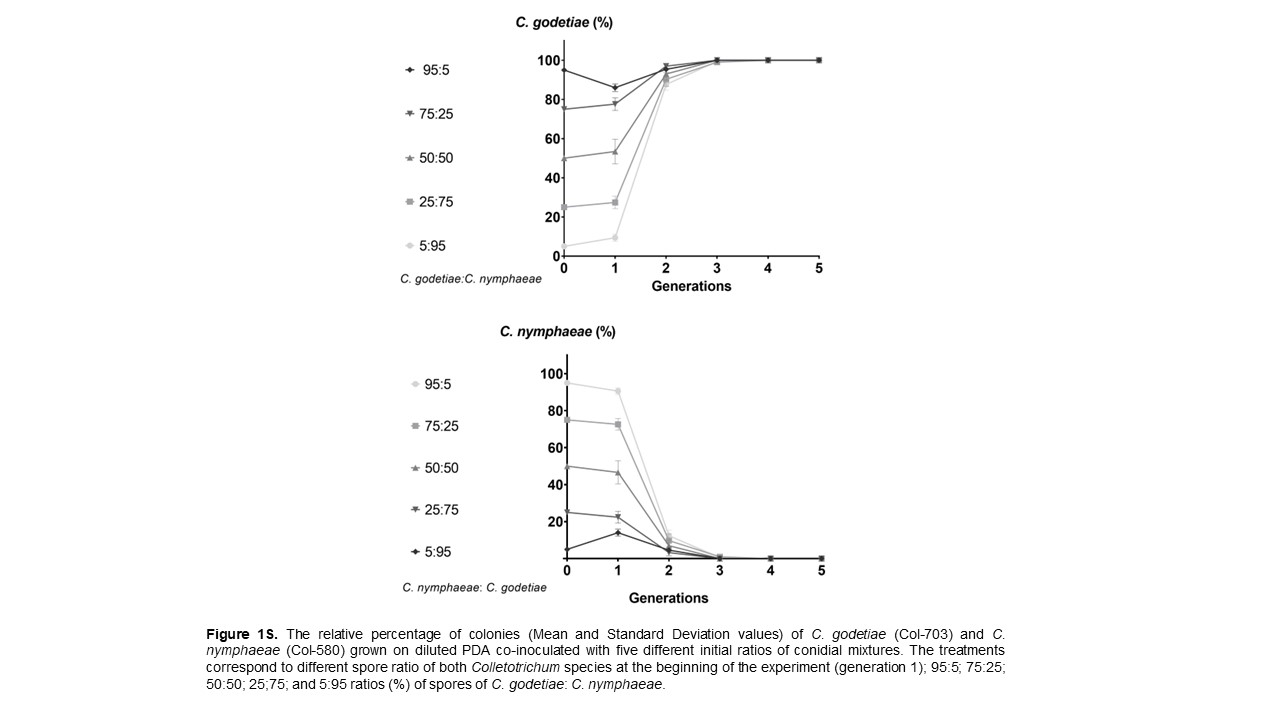

Supplement: Supplementary file 1 — Supplementary Figure 1. [file 41598_2023_32585_MOESM1_ESM.jpg]

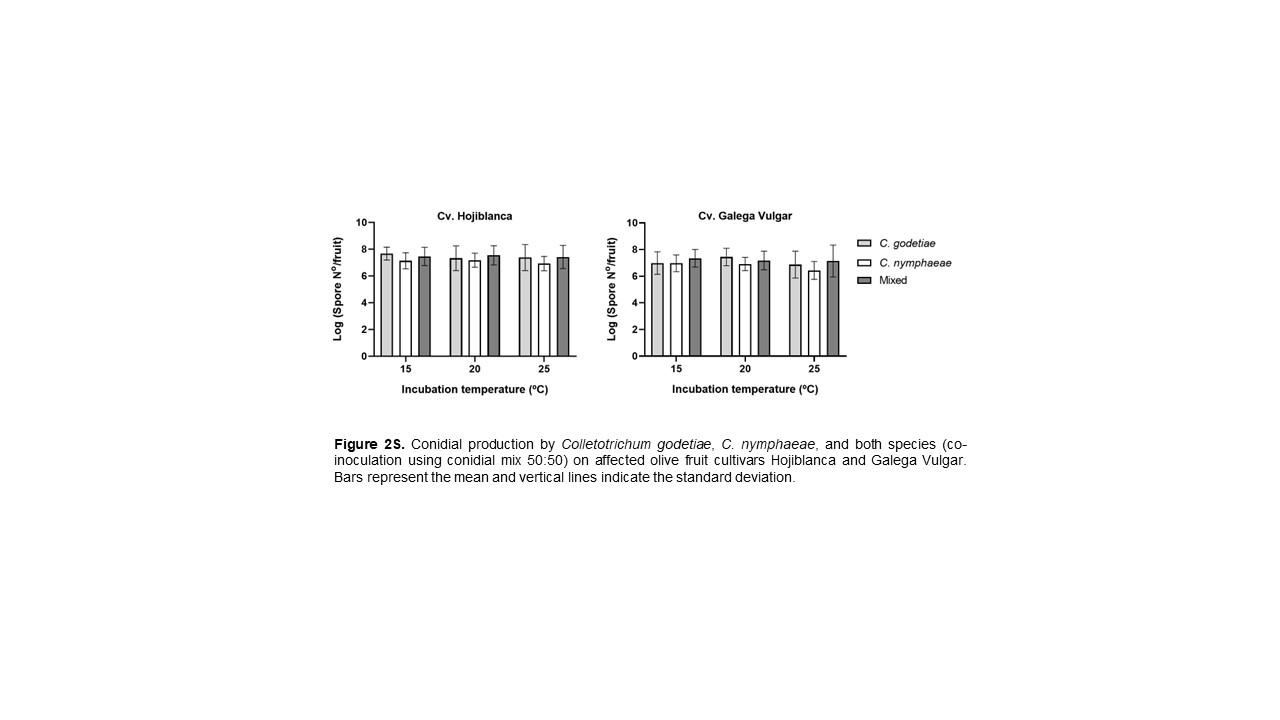

Supplement: Supplementary file 2 — Supplementary Figure 2. [file 41598_2023_32585_MOESM2_ESM.jpg]
